# The Digestive System and Nutritional Considerations for Individuals with Rett Syndrome

**DOI:** 10.1100/tsw.2006.264

**Published:** 2006-12-28

**Authors:** Meir Lotan, Lilit Zysman

**Affiliations:** ^1^ National Evaluation Team, Israel Rett Syndrome Center Chaim Sheba Medical Center and Chaim Sheba Medical Center, Tel HaShomer, Ramat Gan, Israel; ^2^ Department of Physical Therapy, Academic College of Judea and Samaria, Ariel, Israel

**Keywords:** Rett syndrome, intellectual disability, gastrointestinal disease, Israel

## Abstract

Rett syndrome (RS) is a neurodevelopmental syndrome of genetic origin that mainly affects females. Individuals diagnosed with RS exhibit a variety of functional difficulties that impair their quality of life. One of the affected systems is the digestive system, where 74% of persons with RS have abnormal functioning. The affected digestive system causes this population to present an array of problems, such as gastroesophageal reflux (GER), constipation, and malnutrition, leading to failure to thrive (FTT), which resolves in reduced functional ability. Due to the severe effects of the dysfunctional digestive system of individuals with RS, this article will describe the problems common to this population, as well as propose some clinical suggestions for intervention. .

## INTRODUCTION

Rett syndrome (RS) is a genetic disorder that primarily affects females[1,2]. The disorder causes a neurological and developmental arrest that manifests itself in a variety of disabilities, such as loss of functional hand use, loss of acquired speech, apraxia, ataxia, autonomic system dysfunction, epilepsy, breathing abnormalities, failure to thrive (FTT), and muscle tone irregularities[3,4,5]. Swallowing is a complex process that involves a sequence of intricately timed maneuvers executed by a large number of muscles (including muscles of the mouth, pharynx, larynx, esophagus, and diaphragm). Therefore, it is not surprising that this choreography is profoundly disturbed by muscle weakness, dystonia, as well as poor coordination in neuromuscular conditions, such as RS[6].

Digestive system dysfunctions are a significant component of the different phenotypic expression of individuals with RS and at least one gastrointestinal problem can be found among 74% of individuals with this diagnosis[7]. It is a known fact that inadequate food intake and bowel management holds severe consequences for this population[7,8,9,10,11], and due to their poor nutritional state, nutritional intervention is an important element in the daily management of individual with RS. Good nutritional management can improve not only the weight of the individual with RS and her rates of progression on growing charts, but also her activity level as well as her functional abilities[10,11], thereby, positively effecting her overall quality of life.

It is clear that other characteristics of this population, such as abnormality of the respiratory system, (e.g., apnea and hyperventilation episodes)[12] as well as orthopedic difficulties (e.g., muscle tone abnormalities, scoliosis, and postural malalignment)[13,14,15] aggravates the dysfunctional digestive system. All the above-mentioned elements should be addressed by a multiprofessional team approach[16], and such a team should preferably include a pediatric dietitian specializing in developmental disabilities. Due to the fact that the overall functional and health situation of the individual with RS is dependent on her nutritional status, the significance of such a position (pediatric dietitian) is substantial. The dietitian should check the initial state of each client, set appropriate intervention programs, and follow the implementation of these programs in order to ensure that the individual achieves her predetermined weight[17]. Since findings imply that the eating abilities of each individual correspond with her general physical condition[18], the achievement of proper and stable dietary management for the individual with RS sets the base[8] for healthier living. A well-established regime enables her to participate fully in learning experiences as well as to help her flourish without setbacks resulting from a poor nutritional state. Many individuals with RS develop eating problems at early ages, although, 82% are showing such problems at relatively older ages (8–12.5 years)[18]. These findings suggest that early multidisciplinary evaluation of the eating abilities and nutritional state, as well as continuous follow-up of the individuals with RS, should commence at an early age and continue with the aging individual.

The next section of the article will outline some of the common problems attributed to the digestive system of the individual with RS, such as difficulties in eating and swallowing, inappropriate gag reflex, hypersensitivity around the mouth, voluntarily food emitting, breathing irregularities, gastroesophageal reflux (GER), and constipation, and will suggest some intervention methods that have been found appropriate for this population.

## DIFFICULTIES IN EATING AND SWALLOWING

Despite their excellent appetites, individuals with RS commonly present an array of eating problems[18]. Only a few of them actually develop mature chewing patterns and up to 64% of individuals with RS show moderate to severe oral-motor dysfunction[18]. Out of those showing oral-motor dysfunction, 41% of the cases originate from factors influencing chewing, such as:

- Abnormal tongue movements (those, especially towards the left side, were noted in all individuals at stage III–IV of RS), usually caused by lack of movement in the central and frontal area of the tongue[19]
- Hypo- or hypermuscular tone of the tongue
- Poor posture of the spine at the cervical and upper torso areas
- Muscular rigidity of the shoulder girdle (usually exhibited by elevated shoulders)
- Hyperextension of the neck, usually accompanied by
- Tongue thrust[20]

One of the expressions of an oral-esophageal problem is the spitting or vomiting of food. One of the main causes for the spitting/vomiting of food is an inappropriate gag reflex.

### Inappropriate Gag Reflex: Spitting/Vomiting

The gag reflex is usually located in the last third of the mouth, e.g., the soft palate area, in people without neurological problems. Abnormal gag reflex was found present in 20% of individuals with RS[20]. The reflex is produced by internal stimulation, such as sickness, as well as external stimulation, such as pressure or weight (from different foods and substances inside the mouth). Some individuals with RS are unable to elicit an appropriate gag reflex and may feel uncomfortable as a result of stimulation of the area forcing them to vomit. An opposite, even more serious, problem manifests it self when the individual is unable to cough, despite gag reflex stimulation, leaving the bronchioles of the eater with RS defenseless. A change of position forward or backward may cause movement of the food in the mouth, as well as positively affect the pattern of movement of the head and shoulders (e.g., the pattern of movement and the regulation of a high tone), thus reducing the gag reaction.

### Hypersensitivity Around the Mouth

The sensory system of individuals with RS was never thoroughly investigated; nevertheless, accumulating reports suggest that this system does not function normatively in this population[21]. The individual with RS may spit hot or cold food items due to hypersensitivity in the mouth area, or as a result of too large lamps within a serving. Such problems may be addressed through appropriate sensory stimulation around and inside of the mouth. Nonetheless, there are different reasons for spitting besides hypersensitivity.

### Voluntarily Food Emitting: Spitting

In general, this is defined as a strong emission of food in a voluntary manner from the mouth. In fact, in some cases, an individual may be defined as an individual who spits even if she did not voluntarily emit. It is a well-known fact that individuals with RS show respiratory irregularities[12] expressed, in some cases, as explosive exhalation. A second reason for apparent deliberate spitting is a situation where the person's mouth is full of food and there is a need to inhale. Such a situation may be resolved through the exhalation of air (with food particles), thereby forcefully spraying food out of the mouth. Our clinical experience has shown that, in some cases, deliberate spitting drives from behavioral causes, due to the enjoyment the individual with RS receives from the attention she gets as the result of such behavior.

## RECURRENT ASPIRATION

The incidence of recurrent aspiration in selected groups of individuals with intellectual or developmental disability (referred for investigation of feeding difficulties) has shown aspiration in 26 and 27%, respectively[22,23]. Individuals with RS present many factors that may interrupt smooth eating and swallowing, such as apraxia, ataxia, and breathing abnormalities[3]. Abnormal breathing patterns, such as apnea, hyperventilation, breath holding, and bloating, are presented by 84% of individuals with RS usually from stage III of the disease[24]. These incidents are apparent during waking hours alone[25] and as they interfere with the complex mechanism of eating, they may cause recurrent aspirations. Thin liquids are particularly prone to be aspirated[26], therefore, one of the ways of preventing aspirations is liquid thickening and the serving of pureed food items. In some severe cases of recurrent aspiration, oral feeding should be stopped, yet for individuals with RS who take so much pleasure in eating, the termination of all oral intake seems very harsh as a solution for preventing aspirations. The first thing to do in such cases would be to try conventional intervention, such as small-volume meals with pureed food and liquid thickening (elaboration on the subject of pureed food can be found in books such as *The Non-Chew Cookbook*)[27].

If surgical intervention, such as a gastrointestinal tubing (GIT)\percutaneous endoscopic gastrostomy (PEG), is required, one should still consider applying small, low-volume "tasters"[28], providing that they do not jeopardize the eater, for the sake of quality of life of the individual with RS.

## GASTROESOPHAGEAL REFLUX (GER)

GER is the return of the stomach content to the upper digestive system. It was diagnosed in about 15–25% of the individuals with RS[7,20]. This phenomenon is caused when the chain of muscular tissue of the upper digestive system (esophageal, larynx, pharynx, and the pyloric sphincter) is dysfunctional. Since the stomach content is acidic, the result of a slight reflux is a bad taste in the mouth and heartburn. However, in severe cases, GER can cause:

- Aspirations — The entry of stomach content into the airways; may cause coughing and in more severe, chronic situations, recurrent lung infections.
- Prolonged exposure of the esophageal tissue to stomach acidity, causing discomfort from the penetration of stomach content through the esophageal outer coating and, as a result, the inflammation of the area.
- Esophageal narrowing — A recurrent, daily damage to the esophagus will cause adhesions in the inflamed esophageal tissue, leading to difficulty in swallowing and the sensation of food stuck in the throat.

Such a situation can be identified if the person with RS is fed by a nasogastric tube, and the insertion of the tube to the stomach gradually becomes more difficult. This phenomenon usually occurs together with increased difficulty in handling solid foods. Other recurrent health problems may accompany esophageal narrowing, such as vomiting with dried-up brown particles (coffee grounds), as well as signs of distress after mealtimes, as well as a constant and gradual decrease in weight, and loss of appetite.

The incidence of GER is quite high among individuals with developmental disability as a group and specifically among individuals with RS. On top of its high incidence, many individuals with RS will find it difficult to express the fact that they are uncomfortable when in distress. The combination of such dangers necessitates the sensitivity and alertness of the caregiving staff to notify the paraprofessional team and the parents, and direct the individual with RS for further evaluation[18]. If conventional intervention has produced no alleviation of the eater's discomfort, the intervention for GER is handled by a pediatrician or a gastroenterologist. Intervention is applied according to the following steps:

- Phase A — Postural changes during and following mealtimes, dietetic changes, and the use of liquid thickeners. This stage is accompanied with the use of acidity neutralizers, such as Losec (Omeprazole), Pepsid (famotidine), Reglan (metoclopramide), and others.
- Phase B — At this stage, the use of prokinetic drugs is introduced. These medications are intended to increase esophageal clearance and enhance the muscular tone of the pyloric sphincter.
- Phase C — If the elevation in dosage of the formerly used drugs is ineffective, surgical intervention such as fundoplication is suggested.

The fundoplication is a relatively simple, invasive procedure that requires general anesthesia and involves the rotation of the stomach around its vertical axis. This procedure is justified in a person with demonstrable reflux, unresponsive to maximal medical management, and showing troublesome symptoms[28]. This leads to spiraling of the esophagus, causing a mechanical obstruction for upcoming stomach content. In some cases, the side effects of such an operation are the shrinkage of the stomach size and reduction of food capacity, leading to longer mealtime durations. Since individuals with RS are usually slow eaters, this will probably go unnoticed.

## GASTROINTESTINAL PROBLEMS

Gastrointestinal problems have an effect on swallowing and on eating speed. For example, reflux and the slow emptying of the stomach will extend mealtimes. Chronic reflux might cause ulcers, bleeding, and pain during mealtime, thereby affecting esophagus/respiratory timing that will, in turn, have a jeopardizing effect on the individual’s health. The slow emptying of stomach content, if later accompanied by lying down, will probably lead to reflux and might lead to the individual’s refusal to be fed. The slow emptying of the stomach caused by the hypoactive parasympathetic system[29] is commonly accompanied by constipation problems and/or fecal intestinal blockage (two well-known manifestations in the digestive system of individuals with RS).

It is obvious that due to the complexity of the oral-esophageal challenges presented by individuals with RS, a thorough evaluation and intervention should be applied by a multidisciplinary team. Such a multiprofessional approach can take into account all the possible difficulties and will address them with a holistic approach. In some cases, a surgical intervention is required, although in many cases, a better posture or a more appropriate handling during mealtimes may positively influence the eating abilities of the individual with RS. Nevertheless, appropriate intervention regarding feeding is beyond the scope of the present article, which will focus on nutrition and the digestive system.

When dealing with body composition the common term used is BMI (body mass index) is a value calculated from an individual's height and weight ($kg/m^2$). It is used as a rough guide to nutritional status[30]. It was found that individuals with RS show average BMI measurements of 17.5[7], when the normative values should be around 21–26[31]. Due to the fact that individuals with RS are so slim and of small stature, any digestive problem can easily cause a rapid deterioration in weight, leading to failure to thrive (FTT).

## FAILURE TO THRIVE (FTT)

FTT is diagnosed in about 85–90% of individuals with RS, worsening with increased age[18,32]. This may be due to the fact that individuals with RS receive 66.9% of the normative daily caloric intake[33]. FTT is a symptom, not a sickness per se, with distinct criteria. It seems that individuals with RS are in such poor nutritional condition that 85% of them fall under the category of moderate to severe malnutrition[34]. Malnutrition subjects the respiratory muscles to catabolism, leading to atrophy, weakness, and reduced lung function[35], as well as increasing bacterial colonization of the airways and reducing resistance to infection[36].

This situation may be present despite long duration of mealtimes. Due to the severity of the situation and the functional implications of malnutrition, it is important that a close eye be kept on the growth charts of the individual with RS and that any reports of decline from the norm should be presented to the gastroenterologist/dietitian/child neurologist. Joint input from dietician and speech therapist is essential, implementing appropriate feeding modification and supplements in some individuals, while others require nasogastric feeding; if this condition persists for long durations, the implementation of a gastrostomy is usually advocated[28]. It was found that in regard to the norm, individuals with RS present a slightly reduced absorption rate of nutrients. Even so, this slight cutback in absorption, combined with a steady reduction in caloric intake, as well as the individual’s difficulty in reporting her hunger[11], eventually leads to a constant state of malnutrition[11,18].

The conventional management of FTT is the introduction of supplementary, high-caloric intake. Increase in caloric intake is essential for individuals with RS presenting malnutrition, and is extremely recommended since it has been found to enhance the individual’s resilience to external infections, extend attention span during educational experiences, reduce agitation, and enhance the individual's physical activity (the participants were more active, trying to crawl and walk)[9,10]. One should seek the advice of a dietitian in order to build a proper diet for the individual with RS as well as to monitor its effects. In order to enhance caloric intake, one may try a high-caloric, high-fat, low-volume diet that follows the guidelines below:

- Increased caloric intake (especially fat). A dietitian should be consulted to include mostly high-fat yet low-cholesterol food items. Neutral high-caloric ingredients include raw tehina and avocado.
- Care should be taken not to include food items that contain high-sugar ingredients that may lead to dental damage.
- Include vitamins and minerals to enhance general health.
- Include additional meals each day (three main meals + three interim meals).

- Serve as many and as much of the food items that the individual with RS is extremely fond of.
- If a food item that the individual with RS likes is a food stuff that may harm the dental system (such as chocolate or soda), an oral-hygiene program should be implemented simultaneously with the new diet.
- Decrease fluid intake during mealtimes.
- Restrict the intake of juices, especially fruit juices.
- Suggest mainly high-caloric liquids (e.g., Osmolite, Ensure).
- Introduce high-fat sauces to each meal.
- During cooked meals, suggest fried food items (such as French fries, Schnitzels/chicken patties, fried eggplants)[8].

Recommended food items are avocado, sausages, butter, meat, whipped cream, margarine, steaks, cream, sour cream, peanut butter, mayonnaise, cream cheese, fish oil, whole yogurt, and French fries. If the diet is ineffective and the individual with RS continues to show a poor nutritional state, there may be a need for a PEG (e.g., the surgical procedure to insert a GIT).

## PERCUTANEUS ENDOSCOPIC GASTROSTOMY (PEG)/GASTROINTESTINAL TUBE (GIT)

Feeding through a PEG is an invasive method that uses a special tube inserted directly through the abdominal wall into the stomach. The tube is inserted following a surgical or an endoscopical procedure. This method is regarded to be extremely safe, presenting low risk to the patient and enables the maintenance of a proper dietetic state. The PEG is held in place by a balloon inflated after the insertion of the tube inside the stomach. All the PEG systems used today are equipped with a series of unidirectional valves, enabling excess pressure to be released in order to prevent it from building up in the client's stomach, causing reflux. The efficiency of the PEG can be initially tested via the use of nasogastric feeding. Such an experiment will enable the observation of the results of tube feeding without the need for a surgical procedure. Reasons for using a PEG may be:

- Prevention of aspiration and recurrent lung inflammations
- Prevention of, or improvement in, a state of malnutrition (in case this change cannot be achieved through oral feeding) and its consequences (e.g., chronic weight loss and recurrent hospitalizations)
- Prevention of dehydration due to multiple vomiting
- Prevention of reflux, thereby preventing its consequences (heartburn, pain, and a threat to the integrity of the esophagus)
- Enabling the easy and precise insertion of different medications, thereby simplifying the monitoring of the individual with RS's medical condition (including such symptoms as constipation, epilepsy)
- Preventing the inconvenience of vomiting
- Changing mealtime in to purely pleasurable quality time
- Reducing the time spent on feeding and using the extra time to achieve other therapeutic goals (e.g., educational or motor activities)
- Simplifying the work of the caregivers
- Enabling the exact monitoring of caloric intake of the individual with RS
- Enabling extra time for the family of the individual with RS, when time-consuming elements are added to the familial frame (e.g., a new job or baby)
- Replacement of food additives and food supplements that were found ineffective in achieving their dietetic purpose
- Preventing challenging behavior that may have developed during long periods of rejection of food and liquids

The insertion of a PEG is a joint decision by the medical staff and the guardians of the individual with RS. Reilly and Cass[18] constructed a simplified, decision-making flowchart that will help the family when deciding on the proper course of action.

The surgical procedure for the insertion of the GIT is short and easily performed. Following this procedure, there is a need to constantly clean the area around the GIT to prevent infections and inflammations. Within the weeks following the operation, excessive gas production is a common side effect that spontaneously disappears. The daily management of the tube is relatively easy, necessitating the cleaning of the inner and outer walls of the tube with water. There is unanimous agreement as to the success of this procedure by all parents of individuals with RS who have completed it. Within the Australian RS population, 12.5% of individuals with RS use such a device[7]. There is a variety of GIT "off the shelf" supplements, answering to the nutritional needs of all clients; most of them are available in liquid form. These supplements are recommended, since they are sterile and their nutritional composition and caloric value are known. It is possible on the other hand, to use ground food that has more emotional and social advantages, although, the drawback lies in the fact that it may be infected, or present anunified texture[37]

## CONSTIPATION

Constipation has been defined as a delay or as a difficulty in defecation, present for 2 or more weeks, sufficient enough to cause significant distress to the patient[38]. This condition is responsible for an estimated 3–5% of physician visits by children[39]. Leonard[7] reports the most common of digestive problems presented by individuals with RS is constipation, affecting almost two-thirds of the Australian RS population. Saavedra, on the other hand, reports that constipation affects 85% of individuals with RS, occurring at least once in their life span[40].

Reilly and Cass[18] suspected that constipation in individuals with RS was caused by several problems, such as lack of physical activity, low muscle tone, diet, medication, small quantities of drinking, and scoliosis. Constipation may be identified even as early as during the child's first or second year of life. Hard, rare, and large defecations are usually common in this population and they tend to worsen with time if left untreated. Besides the findings regarding the underactive parasympathetic system[12,41], no primary pathological or physiological factors were detected within the neural and muscular structures of the intestines and defecation reflexes can be normally elicited[40].

Since individuals with RS do not present serious organic anatomical or physiological cause for constipation, their constipation can be referred to as functional constipation (see explanation below). This means that the appearance of constipation may be aggravated by the behavioral reaction of the individual suffering from it. It was found that approximately 50% of constipated children contract, rather than relax, the external sphincter complex during a defecation attempt[42]. If the individual with RS is to react in the same way and does not wish to defecate, she may tighten the external anal sphincter and squeeze the glutei muscles. These actions can push feces higher up inside the rectal vault and reduce the urge to defecate. If the individual with RS frequently avoids defecating, the rectum eventually stretches to accommodate the retained fecal mass, and the propulsive power of the rectum is diminished. The longer feces remain in the rectum, the harder it becomes. Passage of a hard or large stool may cause a painful anal fissure. The cycle of avoiding bowel movements because of a fear of painful defecation may progress to stool retention and infrequent bowel movements, a condition that is termed functional constipation[39].

Luckily, early intervention may improve the chance for complete resolution of functional constipation[43]. Treatment goals include disimpacting the rectum and then maintaining a regular bowel movement routine. Months of treatment may be necessary before maintenance medications can be weaned[39]. An individual, anticonstipation solution should be adapted to every individual with RS suffering from constipation by a dietitian, taking into account her age, type of constipation, medical history, and food preferences. Immediate solutions to prevent constipation may be:

- Applying a high-fiber diet — Use food sources such as whole wheat, cereal, breads and pastas, fruits and vegetables (preferably fresh, and with the use of the skin). Preferred fruits are plums, pears, peaches, apricots, and dried fruits. In regards to dry food items, the use of beans, peas, bran, and quaker (i.e., popcorn, nuts, and different kinds of seeds) as a source for a high-fiber diet is sometimes recommended, yet the authors believe that the use of these food items could be hazardous due to the known eating and swallowing problems characteristic of this population[18,20] and, therefore, should be avoided by individuals with RS.
- Using large amount of liquids — An amount of about 8–10 glasses per day (including soup and water-based drinks). In order to achieve the recommended amounts of drinking, it is advisable to insert a regular curriculum of drinking during and in between meals.

A rough calculation could be done in the following manner: If the individual's weight is below 23 kg (50 Libras), then the multiplication of her weight by 125 = minimal daily liquid demands. If the individual's weight is above 23 kg (50 Libras), then the multiplication of her weight by 85 = minimal daily liquid demands (Table 1). Every liquid under room temperature is accountable as drinking (ice cream, yogurt, and sour milk). Important factors which should be considered as well are:

**TABLE 1**
**Recommended Water Requirements per Age and Weight**

| Age | Weight of the person | | Optimal quantity | | # of cups per day |
|---|---|---|---|---|---|
| | Kg. | Libras | In OZ | In CC | |
| 1-3 | 13 | 29 | 54 | 1625 | 6.75 |
| 4-6 | 20 | 44 | 71 | 2125 | 8.75 |
| 7-10 | 28 | 62 | 80 | 2400 | 10 |
| 11+ | 46 | 101 | 84 | 2520 | 10.5 |

- Physical activity promotes regular bowel movements and should therefore be a part of an anticonstipation regime.
- A gentle abdominal massage is a simple way to help defecations and should not be ruled out as one of the primary solutions[18].
- A warm bath sometimes helps to relax the tight anal muscles, reducing pain around this area thereby encouraging defecation[44].
- Paraffin oil could be added to the previous suggestions (when taking into account that paraffin oil reduces the effectiveness of the intestines absorption capability).
- Food items that are known for their ability to promote constipation (apple sauce, white rice, cooked carrots, etc.) should be avoided.
- The regular use of laxatives should only be applied under the guidance of a physician and is usually not recommended due to the fact that it could lead to slowing down of intestinal activity, therefore attributing to a need for gradual increase in dosage and so on and so forth[8].

In order to keep track of the bowel movements of the person with RS, to enable continuous and healthy bowel movements as well as to prevent painful constipation, a defecation chart filled out by the residential and educational facility of the individual is recommended[45]. According to this chart, the following steps should be taken as shown in Table 2.

**TABLE 2**

**Management of Daily Bowel Movement in Rett Syndrome**

| Number of Days | Day Count | Action |
|---|---|---|
| A day with defecation | Day 0 | — |
| Next day, no defecation | Day 1 | — |
| Next day, no defecation | Day 2 | Mineral oil |
| Next day, no defecation | Day 3 | Laxatives, preferably natural laxatives, such as Metamucil, Fiberall/konsy/Senokot |
| Next day, no defecation | Day 4 | Enema |

A day with defecation resets the counts back to day 0. Some dietitians recommend that the use of laxatives and enema should be delayed, therefore, they might suggest a sequence on which: Days 2 + 3, the use of mineral oil; day 4, laxatives; and day 5, enema.

## HOMEMADE RECIPES

As part of the research for this chapter, we screened some old correspondence that went over the RettNet a few years back and gathered some homemade recipes that were suggested by parents of individuals with RS and have been found to work. One should bear in mind that even when applying these homemade solutions, the advice of a dietitian should be sought. Also important is to always start with a few changes; only introduce one new item at a time and advance slowly while closely supervising the reaction.

- At least one defecation a day was achieved through the use of the following daily recipe: quick oats with butter and brown sugar (added liquids are mandatory), baby prunes, canned pears, green beans, and prune juice[46].
- A daily dose of the following recipe was recommended (along with physical activity) to help regular bowel movements: a teaspoon of Mylanta (an antigas ingredient), Simethicon (prevents stomach acidity), a large teaspoon of Metamucil or similar fiber in orange flavor served in a large glass of water. Constant stirring is necessary while the person with RS drinks, to prevent the fibers from settling in the bottom of the glass[47]
- One person with RS has been constipation free for 2 years, ever since she started the following diet and after trying many other solutions, due to drinking a 1/3 cup of the suggested recipe daily: 1 cup fiber, 1 cup of cereal, 12 oz. apple juice, 8 oz, pitted prunes, 2 cups of apple sauce, and 1 cup yogurt. Soak the cereal and prunes in the apple juice until they become soft. Process in a food processor until the mixture becomes a puree; add the apple sauce and mix (the reader should pay attention to the fact that apple sauce, a known constipation enhancer, is a major part of the above-mentioned recipe)[48].
- One person with RS presenting severe constipation at a young age has been on a macrobiotic diet, which basically meant: no dairy products, no red meat, no sugar or salt, no citrus or tropical fruit, and no white (processed) flour. The implementation of the diet has been going on for 15 years with only seldom episodes of constipation without the use of any medications[49].
- A milder solution was adopted by a family that reported they could not get their child with RS to drink enough water, making it problematic to add fiber into her diet. This family is using something called liquid calcium-magnesium. The above-mentioned person received a teaspoon of this medication each morning with breakfast[50]. This medication was cleared by the child's physician, yet due to the impact of magnesium on the heart and the known cardiac irregularities of individuals with RS[51], a consultation with a physician familiar with RS is warranted before commencing the use of the above-mentioned medication.

## WARNINGS

1. Fiber can assist in regular defecation, but if it is given without enough water, it can become the worst bowel blocker. Therefore, the use and dosage should be in consultation with a dietitian, and supervising the water quantities consumed by the individual with RS should be done as well.
2. Prunes are natural laxatives and help in the achievement of regular bowel movements, but their active ingredients may cause dependency as much as any medication. Therefore, constant use of prunes should not be pursued as a first option.
3. In patients unresponsive or intolerant to fiber, both osmotic and stimulant laxatives are effective, but the latter must be avoided for long-term use, as they have a potential for adverse effects[52].
4. An enema can help the individual with RS as well as her family to avoid hours and days of suffering from constipation and bowel impaction, but it does cause dependency by the intestines and should not be used too often.
5. If the individual with RS is on different medication for long periods, especially an antibiotic, the natural intestinal bacteria is destroyed, in which case, the artificial insertion of these bacteria (*Acidophilus* and *Bifidus*) is recommended to enhance proper bowel work and nutritional absorption.
6. Some pain killers (such as morphine or codeine as well as other medication) cause reduction in bowel movements, therefore, the use of such drugs should be avoided. The physician familiar with RS should be consulted when initiating new medication.

If constipation is not regularly and successfully treated, it may deteriorate into a bowel impaction. Therefore, if recurrent episodes of constipation are present, consultation with a gastroenterologist is highly recommended.

## BOWEL IMPACTION

When constipation becomes severe, fecal impaction can result with a mass of compressed feces in the rectum and/or sigmoid colon. Standard treatments include various combinations of oral laxatives and enemas and, when or if these fail, manual evacuation is warranted. The symptoms of bowel impaction are:

- Sudden and severe abdominal tension
- Vomiting of gall bladder juices (green or yellow color)
- Appearance of constant, nonrelenting pain and discomfort
- Reduction in defecation (if constipation was not present beforehand)
- Diarrhea of bloody excrements

All the above-mentioned symptoms require immediate medical intervention[40]. The physician may suggest different intervention approaches for this condition, but some conventional techniques include:

- Daily treatment of 1 l of PEG electrolyte solution, given orally at a dose of 1 l daily for up to 3 days. This method is considered highly effective and acceptable treatment for fecal impaction. This treatment was found effective for patients presenting a wide age range and with different functional and ambulatory states. The stools that passed through patients after treatment were generally of large volume and soft, and most patients graded the act of defecation as being easy[53]. Absence of the need to strain at stool can be an important positive safety aspect, particularly in patients who will not voluntarily assist the clearance of stool, such as individuals with RS.
- Severe constipation or fecal impaction can be resolved through an iso-osmotic laxative taken orally, such as Movicol[54].

- Pulsed-irrigation, enhanced evacuation has been found as a simple, quick, and effective treatment for severe fecal impaction[55].

## ADDITIONAL FACTORS

An additional factor that should be taken into consideration is the fact that 87% of individuals with RS have been found with decreased bone calcium and mineral density[18,56]. Osteoporosis has been reported in very young girls and patients with RS due to decreased bone mineral density compared to controls[57]. This finding puts the individual with RS at risk of suffering pathological fractures due to osteoporosis[57]. It is assumed that this phenomenon results from poor bone formation. These findings support the need for routine checkups of bone density of individuals with RS from childhood and the commencing of physical (such as intensive standing and walking programs[58]), nutritional, and medical intervention, when the situation necessitates such course of action.

In some individuals with RS during adulthood, there seems to be a tendency to become overweight[34]. These individuals should be referred to a dietitian and treated appropriately to achieve weight loss and to prevent functional deterioration due to excessive weight.

## CONCLUSIONS

The sound state of the digestive system of individuals with RS has a significant effect over the wellness and quality of life of the client herself, as well as on her caregivers. Moreover, it was found that 43% of individuals suffering from feeding and digestive problems similar to those presented by individuals with RS are prone to suffer from irritable bowel syndrome (IBS), which aggravates the initial state of the digestive system[59].

Most individuals with RS present a very healthy appetite and according to our experience do not usually require unusual nutritional demands when compared to their peers. Nevertheless, the array of challenges presented by the gastrointestinal system of individuals with RS necessitates constant supervision by a multiprofessional team, including a gastroenterologist as well as a dietitian, while regularly:

- Receiving nutritional evaluations
- Performing feeding assessments
- Repeating instructions for caregivers regarding proper nutritional care

Sometimes, the nutritional necessity to enhance the weight of the individual with RS may be found to contradict her need to maintain an active antiosteoporotic nutritional regime or the need to prevent constipation and bowel impaction. Such contradictions should be acknowledged and resolved by the dietitian. Due to the influence of healthy intestines on the quality of life of the individual with RS and her family, as well as the aggravation that may be caused by a dysfunctional digestive system, not to mention the fact that individuals with RS usually lack the ability to specifically pinpoint their level and location of suffering, it is imperative that constant regular care and attention be given to the digestive system of this population.
